# Müller Cell Regulated Microglial Activation and Migration in Rats With *N*-Methyl-*N*-Nitrosourea-Induced Retinal Degeneration

**DOI:** 10.3389/fnins.2018.00890

**Published:** 2018-12-03

**Authors:** Shuai Zhang, Shanshan Zhang, Wenqing Gong, Guopei Zhu, Songtao Wang, Yalin Wang, Michael Halim, Kaidi Wang, Guomin Zhou, Qiong Liu

**Affiliations:** ^1^Department of Anatomy, Histology and Embryology, School of Basic Medical Sciences, Fudan University, Shanghai, China; ^2^Department of Radiation Oncology, Shanghai Ninth People’s Hospital, Shanghai Jiao Tong University School of Medicine, Shanghai, China; ^3^Department of Integrative Medicine and Neurobiology, School of Basic Medical Sciences, Shanghai, China; ^4^Eye & ENT Hospital, Shanghai Medical College, Fudan University, Shanghai, China; ^5^Key Laboratory of Medical Imaging Computing and Computer Assisted Intervention of Shanghai, Shanghai, China

**Keywords:** retinitis pigmentosa, microglia, *N*-methyl-*N*-nitrosourea, Müller cells, crosstalk

## Abstract

During the pathogenesis of retinitis pigmentosa (RP), the roles of retinal microglial cells after activation have not been fully elucidated. Herein, experimental RP was induced in Sprague Dawley rats by intraperitoneal injection of *N*-methyl-*N*-nitrosourea (MNU) at 50 mg/kg, and the effects of MNU on the retinas were evaluated, respectively, by retinal histology and electroretinography recordings at serial time points. Time-dependent and gradual loss of photoreceptor cells, disrupted arrangement of the outer nuclear layer (ONL), and significant reductions in both a-wave and b-wave amplitudes were observed. Morphology changes were observed in retinal microglial cells; meanwhile, with time, the number of Iba1-positive microglia and their infiltration into the ONL gradually increased. Furthermore, physical interaction of microglial-Müller cell processes following microglial activation was observed after MNU injection. In addition, Müller cells increased CX3CL1 secretion, enhanced microglial cell migration, and upregulated the CX3CR1 expression of the latter. Our observations implied that, during the pathogenesis of RP by MNU, microglial cells exhibit a prominent morphology change and Müller cells can induce activated microglia infiltration by increasing secretion of the chemotaxis factor, CX3CL1, and promoting the migration of retinal microglial cells. This novel finding highlights a potential therapeutic target aimed at regulating the microglial response.

## Introduction

As a heterogeneous retinal disorder, retinitis pigmentosa (RP) is one of the most common causes of severe visual impairment in humans, and the prevalence is approximately 1 in 4000, with approximately 2 million persons affected worldwide (Heckenlively et al., 1988; Hartong et al., 2006). It is known that causative gene mutations, cell stress, the inflammatory response, and retinal remodeling are involved in the pathogenesis of RP, but the outcome is photoreceptor cell death, which results in peripheral vision loss and night blindness (Illing et al., 2002). It has been reported that apoptotic, autophagic, or necrotic signaling are responsible for photoreceptor cell death. Nonetheless, the mechanism of photoreceptor cell death has not been fully clarified; thus, no effective neuroprotective intervention has yet been developed for this entity.

In the central nervous system (CNS), including the retina, microglia are the primary resident immune cell population and also the prominent participant in retinal responses to disease, inflammation, and injury (Moalem and Tracey, 2006). There is an increasing amount of evidence suggesting that microglia can play a beneficial role, but can also present a deleterious feature in the CNS. Microglial cells can respond dynamically to stress signals from the surrounding cells and microenvironment and become activated, undergoing morphological and functional changes. Recent accumulating evidence demonstrates that early microglial activation is involved in many neurological diseases of various etiologies, as a contributor to neurodegeneration; late microglial activation can mediate neuroprotection by modifying the functional phenotype from detrimental to protective (Giunti et al., 2014; Franco and Fernandez-Suarez, 2015). Notably, unlike the broad distribution of microglia in the brain, retinal microglia exhibit a stratified distribution correlating with the laminar organization of the retina (Chen et al., 2002). Furthermore, it has also been reported that retinal microglia are highly regulated by the focal microenvironment (Lee et al., 2008; Marchetti et al., 2011; McCarthy et al., 2013). However, during the process of RP, the roles of microglial cells and how their activation could be regulated by other glial cells have not been fully elucidated. A deeper understanding of these issues is likely central to the clarification of the functional importance of inflammatory damage to the retina, and may reveal the mechanisms related to cell death in photoreceptor cells.

In the current study, we aimed to examine the activation and change of retinal microglia in *N*-methyl-*N*-nitrosourea (MNU)-induced RP animal models. Our observations demonstrated that retinal microglia exhibited a prominent morphological change and migration into the ONL during the pathogenesis of RP. We also found evidence for the physical interaction of microglial-Müller cell processes following microglial activation by MNU. These results may provide a cell target for the development of an effective neuroprotective strategy for retinal diseases associated with photoreceptor cell loss.

## Materials and Methods

### Experimental Animals

Sixty 10-week-old male Sprague Dawley rats were obtained from Shanghai SLAC Laboratory Animal Center. They were housed under controlled temperature (22 ± 2°C), relative humidity (55 ± 10%), 12 h light/dark cycle (7:00 a.m. to 7:00 p.m.), and provided with food and water *ad libitum*. Animal use procedures were approved by the School of Basic Medical Sciences Animal Care and Use Committee and were conducted in accordance with the School of Basic Medical Sciences Guide for the Care and Use of Laboratory Animals.

### RP Model

Experimental rats received a single intraperitoneal injection of 1% MNU (60 mg/kg; Sigma-Aldrich, St. Louis, MO) in freshly prepared saline. Control rats were injected with physiological saline. This RP model was characterized in our previous study (Wan et al., 2008). At 0.5, 1, 2, 5, and 7 days after MNU treatment, rats (10 per group) were euthanized with 8% chloral hydrate (0.5 ml/kg) after undergoing electroretinography (ERG) for visual function tests.

### Tissue Preservation and Histology

The animals were deeply anesthetized with 10% chloral hydrate. The superior side of each eye was marked for orientation, and both eyes were enucleated. The anterior segments of the rat eyeballs were removed immediately. The eyecups were fixed by immersion in 4% paraformaldehyde in 0.1 M phosphate buffer (PB, pH 7.4) overnight at 4°C. After cutting of the cornea and the iris, the lenses were removed, and posterior eyecups were dehydrated in 30% sucrose overnight, and then embedded in Tissue-Tek OCT compound (TAKARA, Japan). Retinas were hemisected and fixed in 4% PFA for either 30 min at room temperature or for 50 min on ice. Tissues to be embedded in wax were fixed in 1:3 acetic acid:methanol for 2–12 h. Cryostat sections were cut at 16 μm, and thaw mounted onto Super-Frost Plus slides (Fisher Scientific, Pittsburgh, United States). Hematoxylin and eosin (HE) staining was carried out.

### ERG Recording

ERG recordings were performed at the following time points after MNU administration: 0.5 day (P0.5), 1 day (P1), 2 days (P2), 5 days (P5), and 7 days (P7). Briefly, all rats were adapted to the dark for 1 h before the ERG experiment. Under dim red light conditions, anesthesia was initially induced by intraperitoneal injection of 8% chloral hydrate (0.5 mL/kg) and supplemented with the same anesthetic (0.2 mL/kg) at 45 min intervals. Rats were lightly secured to a stage to ensure a stable position for the ERG recording. Platinum circellus record electrodes were placed on each cornea and a reference electrode was placed subcutaneously between the eyes. A solution of 1% hydroxypropyl methylcellulose was applied to both eyes to maintain corneal hydration. Six to 10 photopic ERG responses were averaged with a stimulus interval of 2 s, and scotopic responses were averaged with a stimulus interval of 60–120 s according to the stimulus intensity. Signals were recorded with band-pass filters of 1–3 Hz. The amplitude of the a-wave was measured from the baseline to the a-wave trough, and that of the b-wave was measured from the maximum a-wave trough to the maximum b-wave peak.

### Immunofluorescence

Briefly, the sections were de-waxed, rinsed in PB, and incubated in 10% normal goat serum in PB for 1 h at 22 ± 2°C to block any nonspecific binding sites. The sections were then incubated with primary antibodies overnight at 4°C. The primary antibodies used in this study included rabbit anti-Iba1 (1:1000, Wako, Japan) and mouse anti-glutamine synthetase (GS) (1:5000; Sigma, United States) antibodies. The sections were then washed three times in PB and incubated in Cy3-conjugated donkey anti-mouse IgG and fluorescein isothiocyanate-conjugated goat anti-rabbit IgG (Jackson Immuno Research, United States, dilution 1:1000, respectively) for 2 h and the nuclei were stained with DAPI (Sigma, cat. no. D9542, 1:1000). After a thorough washing with PB, the wax-embedded tissues were mounted with fluorescent mounting medium (Dako corporation). Digital images were acquired using a Leica TCS SP8 microscope (Leica, United States), imported into Photoshop (Adobe Systems, United States), and adjusted for brightness and contrast.

### Confocal Microscopy and Image Analysis of Microglia and Müller Cells in Retinal Sections

Following immunohistochemical staining, the retinal sections were imaged using the Leica TCS SP8 confocal system. (Leica, United States). Multiplane z-series were collected using a 40× or 63×, oil-immersion objective. Each z-series spanned 30 μm in depth, and comprised 30–50 images per series, each spaced 0.6–1 μm apart. Co-localization was quantified using Pearson’s correlation coefficient and the overlap coefficient, which were calculated using Image-pro Plus software.

### Western Blot Analysis

After decapitation, the retinas were isolated and homogenized in ice-cold RIPA buffer (50 mM tris buffer, pH 8.0; 150 mM NaCl; 1% NP-40; 0.5% deoxycholate; and 0.1% sodium dodecyl sulfate [SDS]) for Western blot analysis. The lysate was cooled on ice for 30 min and centrifuged at a speed of 12000 ×*g* for 15 min, and the supernatant was collected. Aliquots of tissue samples corresponding to 30 mg of total protein were separated by SDS-polyacrylamide gel electrophoresis and transferred to a polyvinylidene difluoride membrane (Amersham, Newark, United States). The membranes were blocked for 1 h at 22 ± 2°C in 5% nonfat dry milk in tris-buffered saline with 0.01% Tween. The primary antibodies was mouse anti chemokine (C-X3-C motif) receptor 1 (CX3CR1) (1:1000, Santa Cruz, United States). Peroxidase-conjugated secondary antibodies were used (Pierce, United States). The blot was washed three times, for 10 min each time, and the immunoreactive bands were detected using an Enhanced Chemiluminescence Detection Kit (Amersham, United States).

### Cell Transwell Migration Assay

Microglial cells isolated from C57BL/6 mouse brain tissue and were cultured in Dulbecco’s Modified Eagle’s medium (DMEM) with 10% Fetal bovine serum (FBS); retinal Müller cells were purchased from Procell Biotechnology Corporation (Wuhan, China) and cultured in DMEM/F12 medium with 10% FBS and 1% insulin transferrin selenium (Invitrogen).

For the migration assay, 1 × 10^5^ microglial cells were placed in the upper chamber, and 500 μl of DMEM/F12 media with 10% FBS, 1% insulin transferrin selenium, or a supernatant of retinal Müller cells 48 h after culture were added to the lower chamber. Following 20 h incubation at 37°C, the cells on the upper membrane were removed with a cotton swab. The filter was then immersed in methanol for 15 min at 22 ± 2°C and treated with 0.25% crystal violet stain for 10 min at 22 ± 2°C prior to washing with water. The number of cells that had migrated to the lower side of the membrane was counted.

### Enzyme-Linked Immunosorbent Assay

The level of the chemotaxis factor, chemokine (C-X3-C motif) ligand 1 (CX3CL1), in the supernatant from the cultured retinal Müller cells was determined using respective enzyme-linked immunosorbent assay (ELISA) kits (R&D Systems, MN), following the manufacturer’s instructions. In brief, the culture supernatant from the retinal Müller cells was collected following centrifugation at 800 rpm for 10 min and used for ELISA detection. The color changes were determined at 450 nm.

### Statistical Analysis

The data are presented as the mean ± standard error of the mean (SEM) and compared between control and MNU-treated rats. The data were analyzed using a one-way ANOVA and Tukey’s Honestly Significant Difference test. *p* < 0.05 was considered statistically significant.

## Results

### Effects of MNU on the Retinas of Rats Receiving MNU Intraperitoneal Injection

**FIGURE 1:**
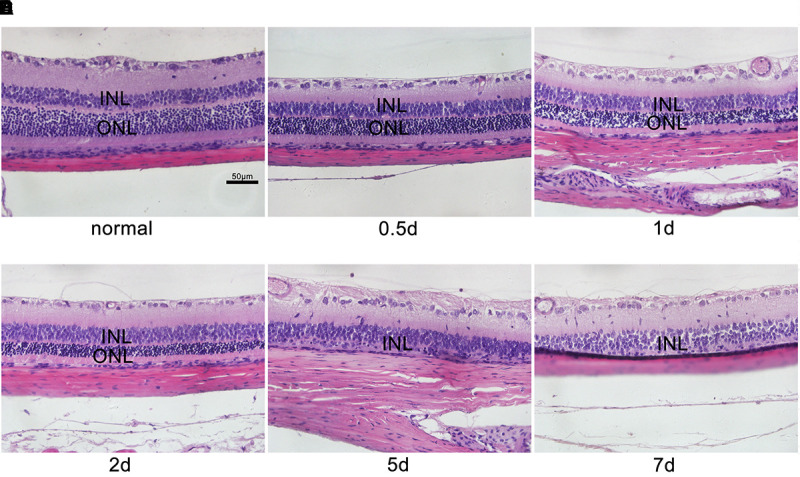
Histological characteristics of *N*-methyl-*N*-nitrosourea-induced retinal degeneration. Retinal sections were reviewed microscopically after hematoxylin and eosin staining at selected time points **(A)** 0 day, **(B)** 0.5 day, **(C)** 1 day, **(D)** 2 days, **(E)** 5 days, and **(F)** 7 days after *N*-methyl-*N*-nitrosourea injection. GCL, ganglion cell layer; INL, inner nuclear layer; ONL, outer nuclear layer. These are central images of the retina, near the optic nerve. Shown are representative photomicrographs (400 × magnification).

**FIGURE 2:**
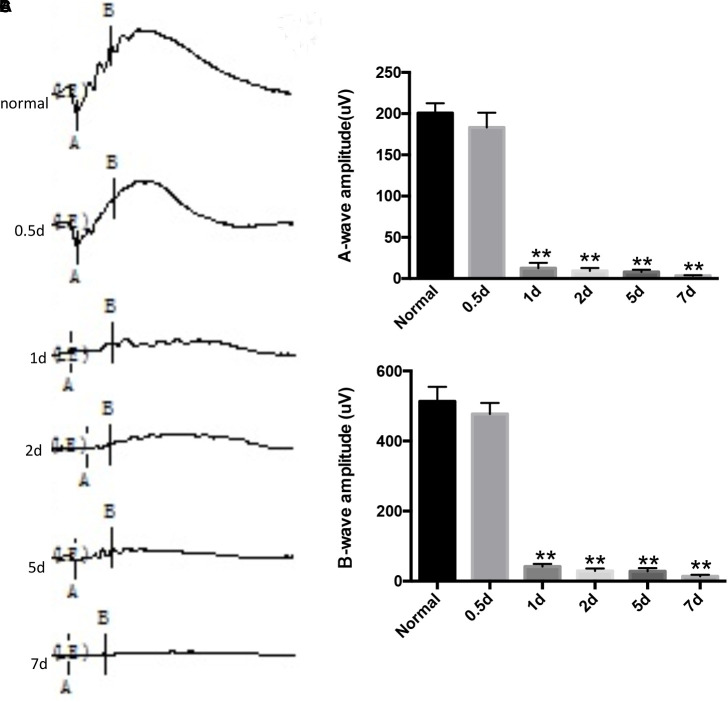
*N*-methyl-*N*-nitrosourea (MNU)-induced retinal function alteration was evaluated by electroretinography (ERG) recordings. The representative ERG waveforms of the normal controls and the MNU-treated retinas at different recording time points and B wave marker position shown is not what was used for quantification in this graph. **(A)**. After MNU administration, the amplitudes of both the a- and b-waves were significantly reduced compared to those of normal controls, as determined by Kruskal–Wallis ANOVA analysis followed by *post-hoc* testing **(B,C)**; ^∗∗^*p* ≤ 0.01 for differences compared to normal controls.

During the experiment, neither death nor clinical signs or symptoms were not observed in rats receiving MNU intraperitoneal injections. The MNU-induced rat RP models were evaluated by retinal histology and ERG recordings at serial time points. In the normal control rat retinas, the outer nuclear layer (ONL) contains 15 strata of well-arranged photoreceptors (Figure 1); in contrast, in the MNU-induced RP rat retinas, time-dependent, and gradual loss of photoreceptor cells and disrupted arrangement of the ONL were observed (Figure 1). In the normal control rat retinas, typical ERG waves were observed (Figure 2). However, significant reductions in both the a-wave and b-wave amplitudes were observed at various time points (P1, P2, P5, and P7), with the exception of P0.5, in the MNU-induced RP rat retinas compared to normal controls (*p* ≤ 0.01); the reduction was particularly evident at P7, at which point an almost undetectable waveform was observed (Figure 2).

### Distribution and Morphology of Retinal Microglia in RP Rat Retinas

**FIGURE 3:**
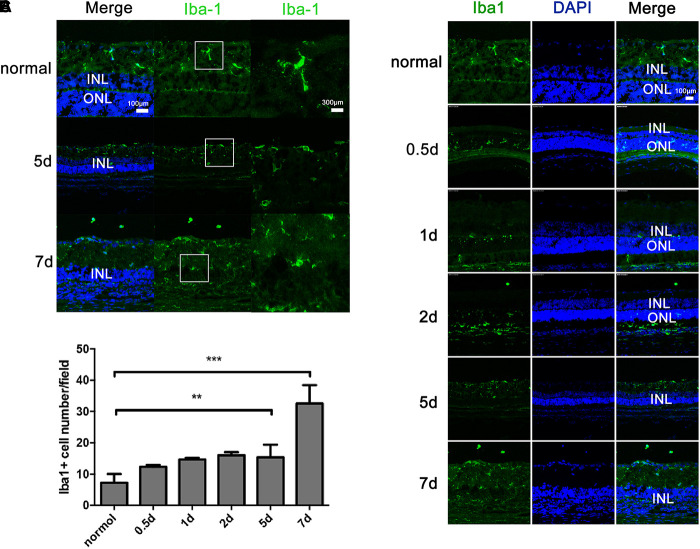
Distribution and activation of microglial cells in rats with *N*-methyl-*N*-nitrosourea-induced retinitis pigmentosa retinal vertical sections were immunolabeled with Iba1 (a unique marker of microglia) and DAPI at selected time points (0.5, 1, 2, 5, and 7 days) after *N*-methyl-*N*-nitrosourea injection **(A,B)**. GCL, ganglion cell layer; INL, inner nuclear layer; ONL, outer nuclear layer. Average number of positively stained microglial cells quantified in whole-mount retinas **(C)**. ^∗∗^*p* ≤ 0.01 for differences compared to normal controls. Shown are representative photomicrographs (1200 × magnification) for A and representative photomicrographs (400 × magnification) for **(B)**. These are central images of the retina, near the optic nerve.

The microglia in the retinas were identified by Iba1 immunoreactivity, a unique marker of microglia/macrophages. In the normal control rat retinas, the microglia were limited to the inner and outer plexiform and ganglion cell layers and appeared as a small cell soma with numerous ramified projections (Figure 3A). In contrast, in the MNU-induced RP rat retinas, an increased number of microglia was observed. The microglia had infiltrated the ONL and were distributed in all retinal layers, and had acquired a rounded, amoeboid morphology with an enlarged soma (Figure 3A). Quantitative analysis demonstrated that, with time, the number of Iba1-positive microglia and their infiltration into the ONL gradually increased (Figures 3B,C).

### Mutual Contraction of Microglial-Müller Cell Processes Following Microglial Activation by MNU

**FIGURE 4:**
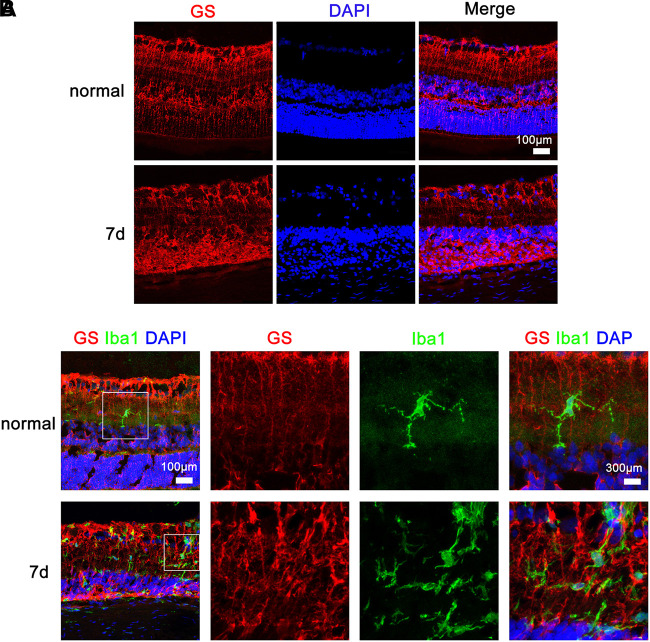
Microglia and Müller cell interaction in the retinas of rats with *N*-methyl-*N*-nitrosourea-induced retinitis pigmentosa immunohistochemical analyses in the retinal sections revealed that the Iba1-positive microglia exhibited overt ramified morphology interdigitated with glutamine synthetase-positive Müller cells with radial-oriented morphology in the inner nuclear layer (INL) before *N*-methyl-*N*-nitrosourea (MNU) injection (normal group); after MNU injection, the retinal microglia exhibited a less-ramified morphology, tended to be radially distributed across the retinal lamina to the INL and the outer nuclear layer (ONL), and were closely fasciculated with Müller cells (7-day model group). Shown are representative photomicrographs (1200 × magnification) for **(A)** and representative photomicrographs (400 × magnification) for **(B)**.

At different time points after MNU intraperitoneal injection, the RP rats were euthanized and their retinas were sectioned for immunohistochemical analysis of Iba1-positive microglia/macrophages and GS-positive Müller cells using confocal microscopy at high magnification. As depicted in Figure 4, in the rat retinas before MNU injection, the Iba1-positive microglia exhibited overt ramified morphology interdigitated with GS-positive Müller cells with radial-oriented morphology in the inner plexiform layer; 7 days after MNU injection, the retinal microglia exhibited a less-ramified morphology, tended to be radially distributed across the retinal lamina to the inner nuclear layer and the outer plexiform layer, and were closely contacted with Müller cells.

### Microglial Migration and CX3CR1 Upregulation Induced by Müller Cells

**FIGURE 5:**
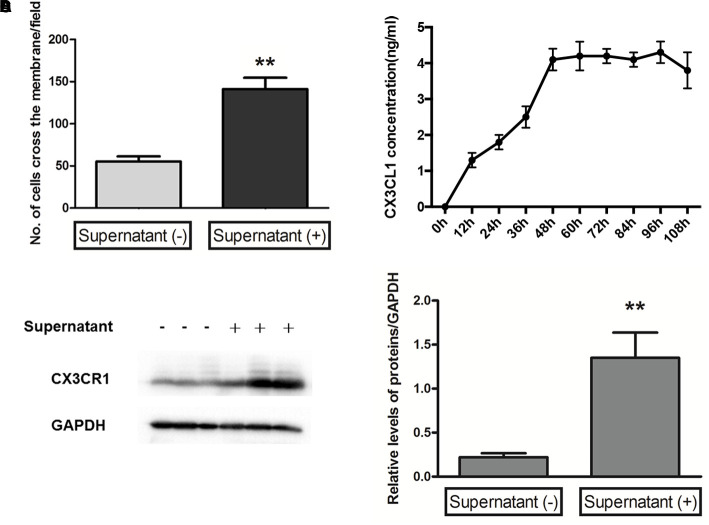
Microglia migration and CX3CR1 expression induced by Müller cells. A transwell migration assay **(A)** determined the microglial cell migration ability in control or supernatant from Müller cells after 48 h in culture, and the number of microglia cells crossing the chambers was analyzed as below (data are presented as mean ± SD, ^∗∗^*p* ≤ 0.01). Time-dependent secretion of CX3CL1 from Müller cells was detected by an enzyme-linked immunosorbent assay **(B)**. The protein expressions of CX3CR1 **(C,D)** were measured by Western blot analysis in microglial cells with and without supernatant from Müller cells after 48 h in culture. Glyceraldehyde-3-phosphate dehydrogenase was detected as a loading control (^∗∗^*p* ≤ 0.01 with compared to without supernatant).

Microglial cells are the innate first-line defense cells in the CNS, including the retina, and constitute approximately 5–12% of the entire CNS cell fraction (Ginhoux et al., 2013). Considering the origin similarities between brain microglia and retinal microglia, which are derived from myeloid progenitors (Polazzi and Monti, 2010), we investigated the migration ability of primary microglia isolated from the C57BL/6 mouse brain with supernatant from retinal Müller cells after 48 h in culture. The results indicated that the cross-membrane number of microglia with supernatant from retinal Müller cells after 48 h in culture was significantly increased compared to that of microglia with culture medium of Müller cells at 0 h in culture (Figure 5A). Meanwhile, it was found that the level of CX3CL1 in the culture supernatant from retinal Müller cells peaked at 48 h, and then plateaued (Figure 5B). Furthermore, CX3CR1 was upregulated in microglial cells cultured with supernatant from retinal Müller cells (Figures 5C,D).

## Discussion

With regard to visual function impairment, RP patients have poor night vision, slowly progressive peripheral-to-central visual field loss, and, often, an eventual decline in visual acuity, primarily owing to photoreceptor death in the retina, in spite of other genetic heterogeneity (Hamel, 2006). Animal models for RP and retinal degeneration are important tools to gain a better understanding of human RP and to explore potential treatments. As a direct-acting alkylating toxicant, MNU induces photoreceptor cell degeneration by transferring its methyl group to nucleobases in nucleic acids to induce cytotoxicity (Hisano et al., 2016). A single systemic administration of MNU can decrease the ONL thickness, degrade the ERG response, and induce photoreceptor cell death, all of which are signs of retinal degeneration similar to the characteristic symptoms of human RP (Boudard et al., 2009). Herein, using the MNU-induced RP rat model, we demonstrated that, during the pathogenesis of RP induced by MNU, retinal microglia were activated and infiltrated the ONL, and the process could be initiated by retinal Müller cells mutual contraction with microglia and promoting the upregulation of CX3CR1in microglia.

A body of evidence suggests that photoreceptor cell loss in RP is promoted by apoptosis (Chang et al., 1993; Porteracailliau et al., 1994; Doonan et al., 2005), but recent evidence suggests that photoreceptor cell death may result primarily from other cell death mechanisms independently of certain apoptotic factors, such as autophagy and necrosis (Sancho-Pelluz et al., 2008). Despite the fact that these molecular mechanisms have been elucidated to some extent, molecular target therapeutic strategies for RP are currently severely hampered, because the majority of cases require therapeutic strategies that are specific to the individual patient. In contrast, therapies focusing on the resident immune cells responsible for photoreceptor cell survival may be more appropriate, as it has been suggested that different molecular mechanisms eventually lead to identical or similar levels of photoreceptor cell death (Furukawa and Koriyama, 2016).

RP was originally named as retinitis, an inflammatory process of the retina. It is now widely accepted that retinal degenerative diseases including RP are characterized by chronic neuroinflammation (Madeira et al., 2015). Microglial cells in the CNS are considered to play a role in regulating the inflammatory environment. Several studies focusing on the role of microglial cells in CNS diseases have demonstrated that the activation and morphology alteration of these cells, including compatibility changes with a reactive phenotype, contribute to retinal neurodegeneration via excessive production of proinflammatory cytokines and increased oxidative and nitrosative stress (Block and Hong, 2005; Langmann, 2007; Karlstetter et al., 2010). Moreover, activated microglial cells migrate toward the injury site, and morphological alteration is often accompanied by activation (Beynon and Walker, 2012). In our study, it was found that, during the MNU-induced RP retina degeneration, the activated microglial cells increased in number, infiltrated the ONL, were distributed throughout the retinal layers, and acquired a rounded, amoeboid morphology with an enlarged soma. Nevertheless, the functions of activated microglia in retinal degeneration are not yet fully elucidated.

Numerous studies suggest that, during retinal degeneration, microglial cells can interact with Müller cells, and such communication can regulate photoreceptor cell survival by acting as a mediator of neuron-glia crosstalk (Zhao et al., 2010; Li et al., 2012; Ginhoux et al., 2013; Wang and Wong, 2014; Fernandez-Sanchez et al., 2015). Also Müller cells can increase their expression of growth factors, such as GDNF and LIF in response to microglial activation (Dalkara et al., 2011; Wang et al., 2011). It has also been reported that, as an ATP provider, Müller cells establish the glial microenvironment, thereby inducing the regulation of microglial dynamic motility (Wang et al., 2014). The research from Wang et al. (2011) showed that, the cell surfaces of Müller glia were resulted in greater adhesion to microglia and induced significantly higher levels of microglial chemotaxis, such as chemokine (C-C motif) ligand 2 (CCL2) and (C-C motif) ligand 3 (CCL3) following co-culture with activated microglia. Müller glia cells also may play an important role in the guidance microglial migration by the presentation of chemotactic guidance cues (Nakazawa et al., 2007; Rutar et al., 2011) as well as physical cell–cell interactions (Sanchez-Lopez et al., 2004; Tassi et al., 2006). In the current study, mutual contraction of microglial-Müller cell processes following microglial activation by MNU was observed; meanwhile, the infiltration of microglial cells across Müller cells and the retinal lamina was also observed. However, the communication between the two cell types in RP is less well clarified. Therefore, we employed an *in vitro* primary cell culture model of cultured microglia with conditioned medium from Müller cells to investigate the effects of Müller cells on microglial motility, with regard to the molecular mechanism. Intriguingly, the results indicated that Müller cells promoted the motility of microglial cells, possibly via secretion of CX3CL1, upregulation of CX3CR1 expression in microglial cells. Thus, we reasoned that the enhanced motility of microglial cells by Müller cells was elicited via the chemotaxis effect of the former.

There are several limitations in our current study. Among these, the major issue is that the primary microglial cells were not directly isolated from the MNU-induced RP rat retinal tissue due to current microglia extract techniques from the retinal tissue of living animal models (Wan et al., 2008). Moreover, the exact molecular mechanism underlying the promotion of chemotaxis initiation in retinal microglial cells by Müller cells was not investigated here. Nevertheless, our *in vitro* and *in vivo* observations implied that, during the pathogenesis of RP by MNU, microglial cells exhibit a prominent morphology change and Müller cells can promote activated microglia to infiltrate the ONL, which is possibly responsible for photoreceptor cell degeneration via increased secretion of CX3CL1 and upregulation of CX3CR1 expression in retinal microglial cells. This novel finding highlights a potential therapeutic target aimed at regulating the responses.

## Conclusion

In summary, during the pathogenesis of RP by MNU, microglial cells exhibited a prominent morphology change, and Müller cells can promote activated microglia infiltration via increased secretion of CX3CL1 and upregulation of CX3CR1 expression in retinal microglial cells.

## Ethics Statement

All animal experiments were approved by the Experimental Animal Ethics Committee of Shanghai Medical College, Fudan University.

## Author Contributions

QL, GmZ, and KW conceived and designed the experiments. SZ, WG, and SsZ performed the experiments. GpZ, MH, YW, and SW analyzed the data and drafted the paper.

## Conflict of Interest Statement

The authors declare that the research was conducted in the absence of any commercial or financial relationships that could be construed as a potential conflict of interest.
