# Corrigendum: Müller Cell Regulated Microglial Activation and Migration in Rats With *N*-Methyl-*N*-Nitrosourea-Induced Retinal Degeneration

**DOI:** 10.3389/fnins.2020.606486

**Published:** 2020-10-22

**Authors:** Shuai Zhang, Shanshan Zhang, Wenqing Gong, Guopei Zhu, Songtao Wang, Yalin Wang, Michael Halim, Kaidi Wang, Guomin Zhou, Qiong Liu

**Affiliations:** ^1^Department of Anatomy, Histology and Embryology, School of Basic Medical Sciences, Fudan University, Shanghai, China; ^2^Department of Radiation Oncology, Shanghai Ninth People's Hospital, Shanghai Jiao Tong University School of Medicine, Shanghai, China; ^3^Department of Integrative Medicine and Neurobiology, School of Basic Medical Sciences, Shanghai, China; ^4^Eye & ENT Hospital, Shanghai Medical College, Fudan University, Shanghai, China; ^5^Key Laboratory of Medical Imaging Computing and Computer Assisted Intervention of Shanghai, Shanghai, China

**Keywords:** retinitis pigmentosa, microglia, *N*-methyl-*N*-nitrosourea, Müller cells, crosstalk

In the published article, there was an error in affiliations 2, 3, and 4. Instead of affiliations:

2. Department of Integrative Medicine and Neurobiology, School of Basic Medical Sciences, Shanghai, China

3. Eye & ENT Hospital, Shanghai Medical College, Fudan University, Shanghai, China

4. Department of Radiation Oncology, Shanghai Ninth People s Hospital, Shanghai Jiao Tong University School of Medicine, Shanghai, China,

it should be:

2. Department of Radiation Oncology, Shanghai Ninth People's Hospital, Shanghai Jiao Tong University School of Medicine, Shanghai, China

3. Department of Integrative Medicine and Neurobiology, School of Basic Medical Sciences, Shanghai, China

4. Eye & ENT Hospital, Shanghai Medical College, Fudan University, Shanghai, China.

The authors apologize for this error and state that this does not change the scientific conclusions of the article in any way. The original article has been updated.

